# Endoscopic Fenestration of Pseudo Cyst in Acute Pancreatitis

**DOI:** 10.1155/DTE.4.155

**Published:** 1998

**Authors:** Fuminori Yamagishi, Mistuyosi Shimoda, Takashi Sakamoto, Kastunori Tauchi, Kastuo Shimada, Takeichi Goka, Tadashi Bandou, Masao Fujimaki, Ademar Yamanaka

**Affiliations:** 1 The Second Department of Surgery Toyama Medical and Pharmaceutical University School of Medicine 2630 Sugitani Toyama 930-01 Japan; 2 Gastrocentor Campinas University Campinas Brazil

## Abstract

We report a case of pseudo cyst accompanied by acute pancreatitis which was successfully treated by endoscopic cyst-gastrostomy. It had been enlarged recurrently after twice simple needle aspiration under ultrasonic monitoring. Because of the infection of the cyst, rapid and complete drainage was needed. Upper gastro-intestinal endoscopy showed a large bulge of the stomach which was compressed by paragastric pancreatic cyst. Endoscopic ultrasonography revealed that the cyst wall was attached hard with the stomach and there was no vessels between them. Endoscopic fenestration of the bulge was created using papillotome and diathermic snare. The drainage was effective and cyst was decompressed rapidly. The fenestration was closed after the cyst was diminished. Recently the endoscopic cyst-gastrostomy made by cutting linearly or inserting catheter have been reported, however, these treatments sometimes resulted in infection and relapse because of the quick closure of the fistula. When the bulge is large and endoscopic ultrasonogram revealed low bleeding risk, the fenestration may be advisable for effective drainage of longer duration without infection.


*Diagnostic and Therapeutic Endoscopy*, Vol. 4, pp. 155–160
Reprints available directly from the publisher
Photocopying permitted by license only

© 1998 OPA (Overseas Publishers Association)
Amsterdam B.V. Published under license
under the Harwood Academic Publishers imprint,
part of The Gordon and Breach Publishing Group.
Printed in Singapore.

### Endoscopic Fenestration of Pseudo Cyst in Acute Pancreatitis: A Case Report

FUMINORI YAMAGISHI[a,*], MISTUYOSI SHIMODA[a], TAKASHI SAKAMOTO[a], KASTUNORI TAUCHI[a], KASTUO SHIMADA[a], TAKEICHI GOKA[a], TADASHI BANDOU[a], MASAO FUJIMAKI[a] and ADEMAR YAMANAKA[a,b]

[a]*The Second Department of Surgery, Toyama Medical and Pharmaceutical University School of Medicine, 2630 Sugitani,Toyama, 930-01, Japan;* [b]*Gastrocentor, Campinas University, Campinas, Brazil*

*(Received 12 February 1997; In final form 29 September 1997)*

**We report a case of pseudo cyst accompanied by acute pancreatitis which was successfully treated by endoscopic cyst-gastrostomy. It had been enlarged recurrently after twice simple needle aspiration under ultrasonic monitoring. Because of the infection of the cyst, rapid and complete drainage was needed. Upper gastro-intestinal endoscopy showed a large bulge of the stomach which was compressed by paragastric pancreatic cyst. Endoscopic ultrasonography revealed that the cyst wall was attached hard with the stomach and there was no vessels between them. Endoscopic fenestration of the bulge was created using papillotome and diathermic snare. The drainage was effective and cyst was decompressed rapidly. The fenestration was closed after the cyst was diminished. Recently the endoscopic cyst-gastrostomy made by cutting linearly or inserting catheter have been reported, however, these treatments sometimes resulted in infection and relapse because of the quick closure of the fistula. When the bulge is large and endoscopic ultrasonogram revealed low bleeding risk, the fenestration may be advisable for effective drainage of longer duration without infection.**

*Keywords:* Cyst-gastrostomy, Endoscopic therapy, Pseudo cyst, Pancreatitis

### INTRODUCTION

Pancreatic pseudo cysts develop in approximately 2–18% of patients with acute pancreatitis [1]. In about 20% of cases the cyst may disappear spontaneously, but most cysts persist and can lead to complications [2]. These include infection resulting in pancreatic abscess, rupture into retroperitoneal cavity or into the digestive tract, and compression of neighboring organs.

The treatment of pancreatic cysts are mainly surgical procedure and two non-surgical approaches have been used too: percutaneous aspiration or drainage under ultrasonic or computed

* Corresponding author. Tel.: 81-764-34-2281 extension 3033. Fax: 81-764-34-5032. E-mail: yamahito@nsknet.or.jp.

tomography (CT) guided and endoscopic drainage. Recently endoscopic drainage of pancreatic cysts was increasingly reported and its result was more successful than any other conservative therapy. However the gastro-cystic fistula created by cutting lineally has good initial drainage, it is frequently closed rapidly and will be following relapse of a cyst or infection. Recently we experienced a case of recurrent pancreas pseudo cyst who had been received cyst-gastrostomy four years ago. Since the cyst had been enlarged recurrently after twice simple needle aspiration under ultrasonic monitoring, we created a fenestration as a cyst-gastrostomy to keep longer and more complete drainage than linear cutting drainage.

### CASE REPORT

A 54-year-old man was referred for evaluation of epigastralgia and back pain. The patient was admitted to our hospital in June 10, 1996. His past medical history was significant for acute pancreatitis, for which he had undergone cyst gastrostomy four years ago. Family history were unremarkable. Physical examination revealed a tender mass in the left upper quadrant. Laboratory data were as follows: hemoglobin, 12.5 g/dl; hematocrit, 41.1% leukocyte count, 5100/mm$^3$; platelet count, $8.6 \times 10^4$/mm$^3$; total serum protein, 6.3 g/dl; total serum bilirubin, 0.8 mg/dl; direct serum bilirubin, 0.5 mg/dl; serum alkaline

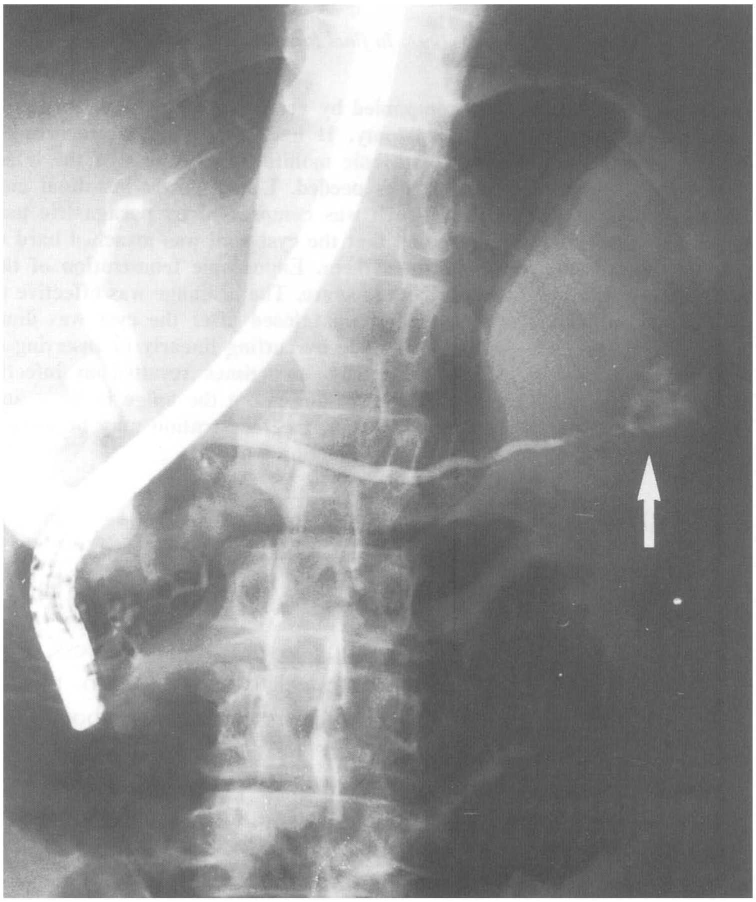

FIGURE 1 ERCP at first admission disclosed a cyst communicating with pancreatic duct.

phosphatase, 269 IU/L; aspartate aminotransferase, 31 IU/L; alanine aminotransferase, 12 IU/L; serum amylase, 99 IU/L. Ultrasonography (US) showed a pancreas tail cyst. CT revealed a cyst at the pancreas tail. Endoscopic retrograde cholangiopancreatography (ERCP) disclosed a cyst communicating with pancreatic duct (Fig. 1). The cyst size was decreased spontaneously and he was discharged from the hospital without surgical treatment. Two months later, he was admitted to our hospital again for recurrent epigastralgia. US and CT revealed a large cyst with thin wall at pancreas tail (Fig. 2). Angiography showed no aneurythm of artery around cyst nor bleeding. Barium meal showed a compression of stomach (Fig. 3(a)). Upper gastrointestinal endoscopy and endoscopic ultrasonography (EUS) revealed gastritis and a bulge of stomach which was compressed by paragastric cyst (Fig. 4(a)). The cyst wall was attached hard with the stomach and there was no vessels between them (Fig. 5). These findings were interpreted as a recurrent pancreatic cyst. Simple needle aspiration under ultrasonic monitoring was performed twice. Although 300 and 660 ml dark-brownish fluid were aspirated by these treatments, the cyst was enlarged recurrently and he was febrile. The infection of the cyst was suspected.

Endoscopic treatment was performed at October 25, 1996. Procedure was as follows. The first step was to coagulate the surface of the bulge by diathermy for hemostasis. Then the bulge was punctured with a needle diathermy. As soon as the needle reached the cyst cavity, the infected fluid escaped into the gastric lumen (Fig. 4(b)). The next step was cyststomy. An papillotome was inserted through the fistula into the cyst cavity and an opening was made like tear shape. Then the fenestration was made by cutting the tear shape flap with a diathemic snare and grasping

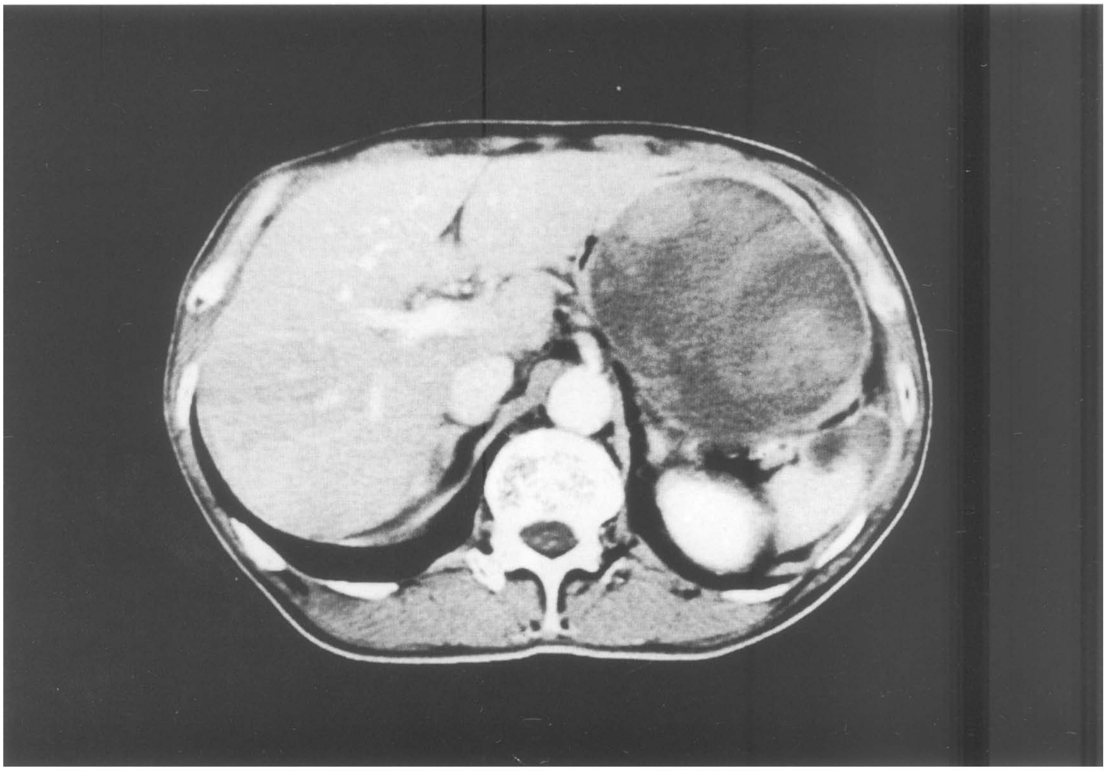

FIGURE 2 CT at second admission revealed a large cyst with thin wall at the pancreas tail.

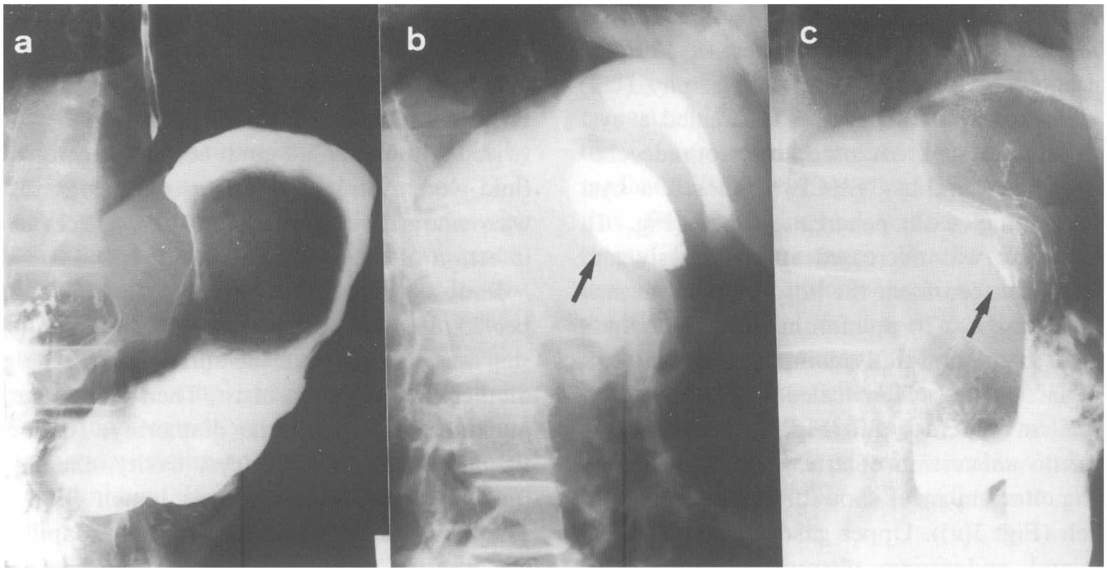

FIGURE 3 (a) Contrast radiograph showed a compression of the stomach before the cyst gastrostomy. (b) A decreased cyst cavity and no leakage of contrast medium right after the cyst gastrostomy. (c) A fistula and following small cavity two weeks after the treatment.

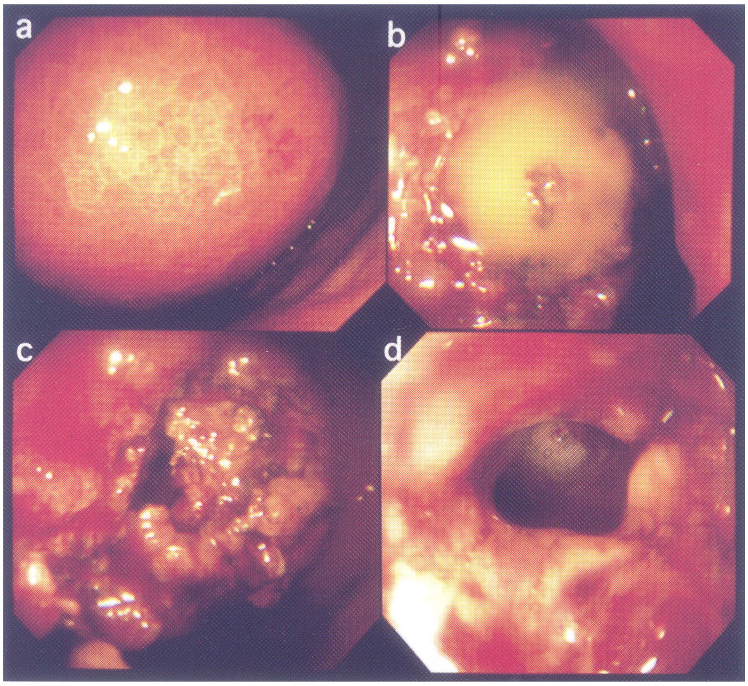

FIGURE 4 (a) Upper gastro-intestinal endoscopy revealed gastritis and a bulge of stomach which was compressed by paragastric cyst. (b) After the needle reached the cyst cavity, the infected fluid escaped into the gastric lumen. (c) The fenestration which was made at the top of the bulge. (d) Endoscopic feature of cyst gastrostomy 7 days after treatment.

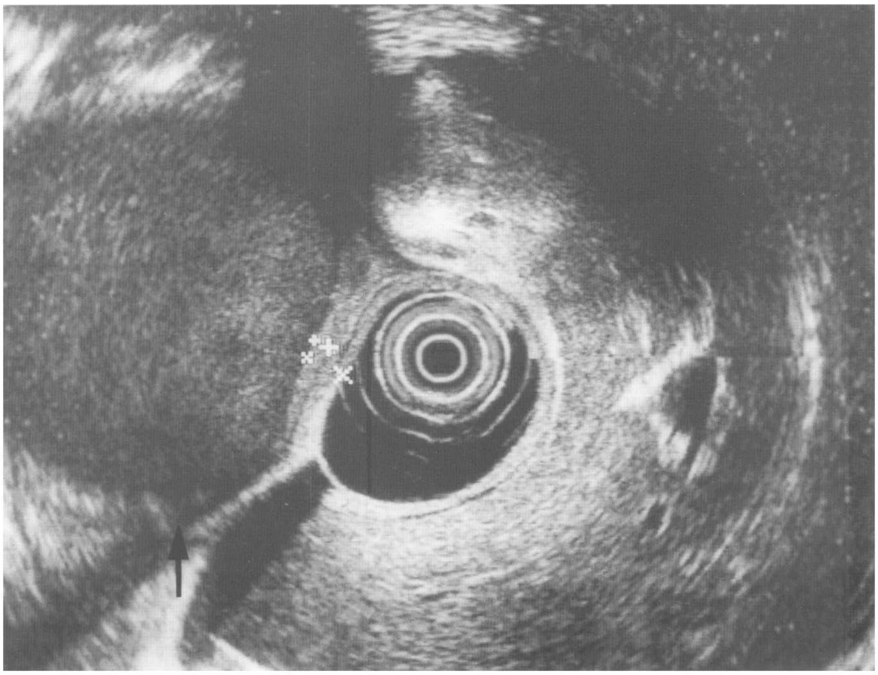

FIGURE 5 EUS showed the cyst wall and stomach which was attached hard, and there was no vessels between them.

forceps. The size of the opening was 20 mm × 20 mm (Fig. 4(c)). Fluoroscopy showed a decreased cyst cavity and no leakage of contrast medium (Fig. 3(b)). There was no complication like bleeding or peritonitis. Gastrointestinal endoscopy was performed one and two weeks after repeatedly. The fenestration was not closed yet and the gastritis was cured (Fig. 4(d)). Fluoroscopy showed the fistula and following small cystic lumen (Fig. 3(c)). CT showed diminished cyst. There was no remarkable complications like abdominal pain, fever, infection or massive bleeding. The patient started eating from 5 POD.

### DISCUSSION

There are several ways to treat pancreatic pseudo cysts. As for surgical treatment, cyst-gastrostomy, cyst-duodenostomy and cyst-jejunostomy are performed for mature cyst which have a thick wall [3]. Although these surgical treatments have been reported good results, immature cyst found in acute phase of pancreatitis is not an indication of cyst-enterostomy because of the relapse of cyst or anastomotic break-down. On the other hand, percutaneous aspiration or drainage under ultrasonic or CT guided and endoscopic drainage have been increasingly performed as conservative therapy. Simple fine needle percutaneous aspiration is initially effective but Grosso *et al.* [4] reported highly recurrence rate (71%). Prolonged extragastric or transgastric external drainage with indwelling catheters is more successful, with 20–25% recurrence rate [1,5–7]. However that procedure may lead to pancreatico-cutaneous fistula or bacterial infection, and problems such as poor quality of life, long hospital stay, delay in rehabilitation, and a risk of accidental catheter pullout. Endoscopic drainage of pancreatic cysts was reported by Rogers *et al.* [8] at first. Then Cremer [9] and Sahel [10] reported endoscopic cyst-duodenostomy and cyst-gastrostomy. They cut the bulging cyst 5–15 mm long linearly with a diathermic papillotome and inserted a drainage catheter in some cases. This endoscopic cyst-enterostomy was technically successful in 90% cases. A reduction of the cyst and pain relief rate were more than 80%. The total relapse rate of cyst-duodenostomy and cyst-gastrostomy was 9–19%. The fistulas of cyst-gastrostomy closed more rapidly than that of the cyst-duodenostomy, the relapse rate of cyst-gastrostomy was higher than that of the latter. To avoid the relapse, the nasogastrocystic catheter were left in place for long time, but it often results in a higher risk of infection.

We made a fenestration at the top of the bulging to keep a long term opening of the drainage without catheter. The drainage was effective and cyst was decompressed rapidly. The fenestration was kept opening after cyst was diminished.

Although there was no remarkable complication in this case, the main risk of this treatment seems to be uncontrolled bleeding. Therefore, EUS, magnetic resonance imaging and angiography to check for potential bleeding from adjacent vessels before this procedure is advisable. EUS was particularly useful for detecting small vessels between stomach and cyst. Additionally we set several facilities to control the bleeding; hemoclip, microwave coagulator. Using these facilities, this method will be done safely as a treatment of pancreatic pseudo cyst.

### References

[1] Torres, W.E., Evert, M.B., Baumgartner, B.R. *et al.* Percutanous aspiration and drainage of pancreatic pseudocysts. *Am. J. Roentgenol.* 1986; **147**: 1007–1009.

[2] Bradley, E.L., Clements, J.L. and Gonzalez, A.C. The natural history of pancreatic pseudocysts: a unified concept of management. *Am. J. Surg.* 1979; **137**: 135–141.

[3] Newell, K.A., Liu, T. and Aranha, G.V. Are cystgastrostomy and cystjejunostomy equivalent operations for pancreatic pseudocysts? *Surgery* 1990; **108**: 635–640.

[4] Grosso, M., Gandini, G., Cassinis, M.C. *et al.* Percutaneous treatment (including pseudocystgastrostomy) of 74 pancreatic pseudocysts. *Radiology* 1989; **173**: 493–497.

[5] Karlson, K.B., Martin, E.C., Frankuchen, E.I. *et al.* Percutaneous drainage of pancreatic pseudocysts and abscess. *Radiology* 1982; **142**: 619–624.

[6] Van Sonnenberg, E., Wittich, G.R., Casola, G. *et al.* Complicated pancreatic inflammatoly disease: diagnosis and therapeutic role of intervational radiology. *Radiology* 1985; **155**: 335–340.

[7] Freeny, P.C., Lewis, G.P., Traverso, L.W. *et al.* Infected pancreatic fluid collections: percutaneous catheter drainage. *Radiology* 1988; **167**: 435–441.

[8] Rogers, B.H.G., Circurel, N.J., Seed, R.W. *et al.* Transgastric needle aspiration of pancreatic pseudocysts through an endoscope. *Gastrointest. Endosc.* 1975; **21**: 133–134.

[9] Cremer, M., Deviere, J. and Engelholm, L. Endoscopic management of cysts and pseudocysts in chronic pancreatitis: longterm follow-up after 7 years of experience. *Gastrointest. Endosc.* 1989; **35**: 1–9.

[10] Sahel, J., Bastid, C., Pellat, B. *et al.* Endoscopic cystoduodenostomy of cysts of chronic calcifying pancreatitis: A report of 20 cases. *Pancreas* 1987; **2**: 447–453.

